# The Evolution of Gene Therapy in the Treatment of Metabolic Liver Diseases

**DOI:** 10.3390/genes11080915

**Published:** 2020-08-10

**Authors:** Carlos G. Moscoso, Clifford J. Steer

**Affiliations:** 1 Department of Medicine, Division of Gastroenterology, Hepatology and Nutrition, University of Minnesota Medical School, Minneapolis, MN 55455, USA; 2 Department of Genetics, Cell Biology and Development, University of Minnesota Medical School, Minneapolis, MN 55455, USA

**Keywords:** genome editing, liver-targeted gene therapy, CRISPR/Cas9, TALENs, zinc finger nucleases, viral vectors

## Abstract

Monogenic metabolic disorders of hepatic origin number in the hundreds, and for many, liver transplantation remains the only cure. Liver-targeted gene therapy is an attractive treatment modality for many of these conditions, and there have been significant advances at both the preclinical and clinical stages. Viral vectors, including retroviruses, lentiviruses, adenovirus-based vectors, adeno-associated viruses and simian virus 40, have differing safety, efficacy and immunogenic profiles, and several of these have been used in clinical trials with variable success. In this review, we profile viral vectors and non-viral vectors, together with various payloads, including emerging therapies based on RNA, that are entering clinical trials. Genome editing technologies are explored, from earlier to more recent novel approaches that are more efficient, specific and safe in reaching their target sites. The various curative approaches for the multitude of monogenic hepatic metabolic disorders currently at the clinical development stage portend a favorable outlook for this class of genetic disorders.

## 1. Introduction

The liver is the principal organ in the body involved in the metabolism and detoxification of numerous agents; and multiple disorders of metabolism are of hepatic origin. There are as many as 400 inherited and acquired metabolic disorders caused by a single gene mutation, and for many of these, orthotopic liver transplantation is the only known curative therapy. Liver-targeted gene therapy (LTGT) has been successfully applied in preclinical models of various disorders, including hemophilia A and B, urea cycle disorders and familial hypercholesterolemia, with varying degrees of success. Given the monogenic nature of many of these disorders, a gene therapy approach appears naturally suited for therapeutic benefit. Viral vector-based approaches have overcome significant hurdles, with progress made on inherent viral protein immunogenicity, toxicity, specificity of target site delivery and methods of delivery. In addition, non-viral approaches, including genome editing, have made significant advances in improving the safety and efficacy profile of these novel technologies to gene therapy. In conclusion, we present in this review the evolution and current status of the various technologies available for LTGT as well as the challenges that still exist.

## 2. Viral Vectors

### 2.1. Retroviral and Lentiviral Vectors

**Figure 1:**
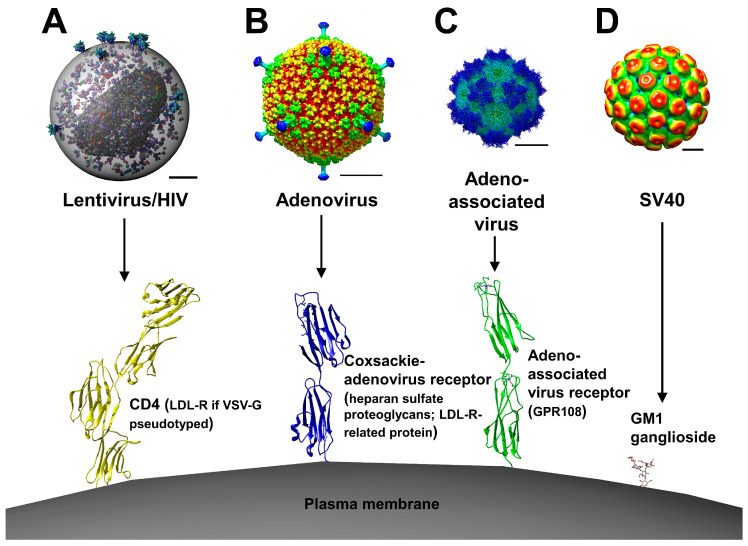
Viral vectors used in liver-targeted gene therapy. (**A**) Lentiviral vectors are able to transduce dividing and non-dividing cells and have their origin in HIV (rendered as cellPACK molecular model) [86]. HIV-based vectors bind to CD4 receptors (PDB ID: 1WIO) [87] as well as to CCR5 or CXCR4 coreceptors, mediating entry into the host cell via a cell–viral membrane fusion mechanism. Several lentiviral vectors are pseudotyped with vesicular stomatitis virus glycoprotein (VSV-G), as well as envelope glycoproteins of other vesiculoviruses, the cognate receptor of which is the low-density lipoprotein receptor (LDL-R) [16,17]. Scale bar = 300 Å. (**B**) Adenoviruses (EMDB ID: EMD-5538) [88] enter cells via attachment to the Coxsackie adenovirus receptor (CAR) (PDB ID: 3JZ7) [89], then attachment to αvβ3/5 integrins. Receptor attachment triggers clathrin-mediated, dynamin-dependent endocytosis, with subsequent endosomal escape, cytosolic transport via microtubules and DNA nuclear import via engagement with the nuclear pore complex (NPC). Adenoviruses can also enter the cell via CAR-independent mechanisms by binding to factor XI and factor X, then subsequent cellular binding to heparan sulfate proteoglycans (HSPGs) or the LDL-R-related protein [90]. Scale bar = 300 Å. (**C**) Adeno-associated viruses (EMDB ID: EMD-9012) [91] attach to cells first via HSPGs, then by engagement with the adeno-associated virus receptor (AAVR) (PDB ID: 6NZ0) [92], subsequently entering the cell via a clathrin-mediated or caveolin-mediated endocytosis mechanism, although AAVR-independent mechanisms have been postulated. More recently, a highly conserved G-protein coupled receptor, GPR108, was identified, which plays an important role in viral entry and nuclear localization [93]. Virions are then trafficked via endosomes to the trans-Golgi network, ultimately leading to escape into the cytoplasm and engagement with the NPC for nucleoplasm mobilization, followed by partial uncoating and genome release. Productive cell infection is dependent upon concomitant adenovirus or herpesvirus infection. Scale bar = 100 Å. (**D**) Simian virus 40 (SV40) (EMDB ID: EMD-5187) [94] binds to the cell membrane via attachment of its surface VP1 pentamer to GM1 ganglioside (PDB ID: 3BWR) [95], with subsequent caveolin-dependent endocytosis and targeting the endoplasmic reticulum (ER) via endosomal trafficking. In the ER, viral capsid destabilization via the reduction of disulfide bonds leads to ER membrane penetration and viral escape into the cytosol. It is unclear whether the viral capsid disassembles prior to or after engagement with the NPC. Scale bar = 100 Å. Virus–receptor interactions are not to scale. All images rendered using UCSF Chimera [96].

As their name implies, retroviruses reverse-transcribe their RNA genome into DNA, which subsequently forms part of the pre-integration nucleoprotein complex. Retrovirus-based vectors, based principally on murine leukemia virus (MLV), have been used in multiple clinical trials of gene therapy (Figure 1A). Retroviral vectors with modified and pseudotyped envelope proteins have been engineered to selectively target different cell types, including hepatocytes [1,2,3,4]. Since hepatocytes are largely quiescent and do not replicate frequently in vivo, the use of retroviruses in LTGT is largely limited to lentiviruses, given that MLV pre-integration nucleosome complexes are unable to cross the nuclear membrane [5]. Notwithstanding this limitation, an in vivo murine study showed stable correction of hemophilia A via retroviral expression of human factor VIII [6]. However, the development of leukemia following the clinical use of retroviral vectors in ex vivo hematopoietic stem cell therapy for severe combined immunodeficiency disorder [7] exemplified the insertional oncogenic risk associated with retroviral vectors.

Lentiviral vectors are derived from HIV and have been well characterized in the literature for their ability to transduce quiescent cells [8]. LTGT is an attractive application compared to retroviruses given their improved stability [9], ability to transduce quiescent cells [10,11], improved titers [12] and reduced frequency of insertional mutagenesis [13,14,15]. While lentiviral vectors are often pseudotyped with vesicular stomatitis virus glycoprotein (VSV-G) for preclinical studies [16,17], the finding that human complement inactivates these vectors and lowers their transduction efficacy [18] has resulted in the use of complement-resistant vesiculovirus strains for pseudotyping [19]. Integration of the human phagocytosis inhibitor CD47 into lentiviral particles via budding in cell lines overexpressing CD47 resulted in improved gene transfer, enhanced hepato- and splenoselectivity, and reduced insertional mutagenesis. This allowed the use of lower doses of lentiviral administration to achieve supraphysiologic factor IX levels in a nonhuman primate model of hemophilia B [20]. Integrase-defective lentiviral vectors resulted in sustained transgene expression, and the prevention of the induction of neutralizing antibodies against factor IX was observed even after re-challenge in a murine model of hemophilia B [21]. HBV (hepatitis B virus) replication was inhibited using CRISPR/Cas9 (clustered regularly interspaced palindromic repeats/CRISPR-associated protein 9) via lentiviral transduction in vivo and in vitro [22,23]. A Phase I trial is currently underway for hemophilia B, using patient stem cells, the ex vivo gene correction of factor IX using an advanced lentiviral vector and autologous gene therapy (ClinicalTrials.gov identifier NCT03961243). The incorporation of protective elements to improve lentiviral transduction and transgene expression, such as CD47 and integrase-defective variants, are important advances that render lentiviral vectors more attractive for future gene therapy clinical trials.

### 2.2. Adenoviral Vectors

Adenoviruses are enveloped double-stranded DNA viruses capable of infecting both quiescent and dividing cells (Figure 1B). There are concerns with the persistence of gene expression following LTGT using adenoviral vectors, given that as much as 95% of gene expression is lost over the course of one year in murine models [24]. Though it has been shown that the mechanism for the loss of gene expression is not solely due to cellular division [25], the exact mechanisms for both residual expression and reactivation are poorly understood. A rat model of Crigler–Najjar syndrome was used to correct hyperbilirubinemia via the hepatic delivery of an adenoviral vector encoding uridine diphosphate glucuronosyltransferase isoform 1A1 (UGT1A1), the enzyme that conjugates bilirubin [26]. In a rat model of estrogen-induced cholestasis, the human *aquaporin 1* gene was delivered via an adenoviral vector and resulted in a lower serum concentration of bile salts and improved biliary output [27]. Transgenic mice with a deletion of miR-221/222 were experimentally challenged to develop non-alcoholic steatohepatitis (NASH). Reintroduction of miR-221/222 via an adenoviral vector controlled the expression of target gene *Timp3* and promoted the progression of NASH in a murine model [28].

Adenoviral vectors are beset by widely prevalent pre-existing immunity, which can limit the effectiveness of adenovirus-based vaccines and gene therapy [29,30]. Specifically, adenovirus-specific CD8^+^ T lymphocyte-mediated cytotoxicity against adenovirus-infected cells caused cell lysis and the loss of transgene, reducing the efficacy of expression [31]. This risk has been attenuated by the engineering of “gutless” rAd vectors, termed helper-dependent adenovirus (HDAd), with some moderate success in vivo [32]. The small size of the early region 1 genes (*E1*) limits the size of the transgene that can be used in first-generation adenoviral vectors, as *E1* is replaced by the transgene. HDAd vectors circumvent this problem by removing all viral genes from the vector, leaving only *cis*-acting elements needed for vector genome replication and encapsidation, and freeing up to 37 kb of space for large transgenes [33]. HDAd vectors elicited minimal cytotoxic T lymphocyte responses due to a lack of expression of viral proteins in transduced cells [34]. They are, as their name suggests, dependent on a helper adenovirus for propagation given the lack of expression of viral proteins needed to replicate and to package the HDAd vector. The adenovirus genome mostly does not integrate and remains episomal, with integration being reported at low frequency [35] and, thus, there is minimal risk of insertional mutagenesis [36]. The long-term expression of transgenes has been reported in animals [37,38]. This system has been successfully used in LTGT. Co-transduction of HDAd with *Sleeping Beauty* transposons in a canine hemophilia B model resulted in the stable expression of factor IX for nearly 1000 days [39,40]. In a mouse model of primary hyperoxaluria 1 (PH1), the increased stable expression of alanine:glyoxylate aminotransferase (AGT) and decreased hyperoxaluria were observed, thus establishing the long-term correction of PH1 [41]. The use of HDAd vectors in a nonhuman primate model of α-1 antitrypsin (AAT) deficiency resulted in the stable expression of AAT [32]. There have not yet been any successful clinical applications using HDAd vectors and contamination with immunogenic helper adenovirus can lead to acute genotoxicity and the induction of severe immune responses in the host, raising concerns about the safety of this approach.

### 2.3. Adeno-Associated Viral Vectors

The adeno-associated virus (AAV) is a single-stranded DNA, non-enveloped, replication-defective virus (Figure 1C) [42]. Productive infection with a lytic phase only occurs in the presence of a helper virus, canonically either herpesvirus or adenovirus, with complex interactions and contributions to the AAV life cycle. In the absence of a helper virus, there is limited replication and viral gene expression. AAV serotype 2 (AAV2) has been shown to integrate specifically in chromosome 19q13.4 (the AAVS1 locus) [43] and establish latency in the presence of Rep viral proteins and via interaction with inverted terminal repeats. In humans, up to 90% of the population has been exposed to AAV2, and thus recombinant AAV (rAAV) vectors are safer and the most frequently used viral vectors for LTGT. A significant proportion of exposed humans develop neutralizing antibodies against AAV, with high cross-reactivity between serotypes, which impacts the utility of AAV vectors [44]. Indeed, in one AAV clinical trial, all subjects developed neutralizing antibodies against the vector capsid [45]. As such, the detection of neutralizing antibodies is important prior to preclinical and clinical studies, as is the monitoring of humoral and cellular responses following administration. Up to 96% of the AAV genome can be replaced with transgenic DNA and while AAV2 is the most widely used serotype, other serotypes have been shown to have differential tropism and transduction efficiency [46]. For example, serotype 3 (AAV3) has been identified as a promising serotype for transduction in human hepatocytes and hepatocellular carcinoma (HCC) cells [47,48]. AAV expression is primarily episomal (extrachromosomal) in nature [48]. While this may suggest that AAV would have suboptimal transduction efficiency in dividing cells, various serotypes have been shown to transduce dividing and non-dividing cells. Specifically, AAV2, AAV3 and AAV6 are able to transduce HEK293, HeLa, HBEC and Saos-2 cell lines efficiently [46]. A mouse study of hemophilia B found that the expression of factor IX administered via an rAAV vector decreased by 92% following two-thirds hepatectomy, suggesting limited expression of the episomal transgene with cell replication [48].

Chimeric AAV vectors have been recombinantly engineered to combine favorable qualities of various AAV serotypes. For example, the AAV-DJ recombinant vector is a chimera derived from serotypes 2, 8 and 9 via a DNA family shuffling approach [49]. AAV-DJ was superior to eight standard AAV serotypes for in vitro transduction, mediated robust human factor IX expression in mice and a heparin-binding domain limited distribution of this vector largely to the liver. More recently, a chimera comprising seven different AAV serotypes (1, 2, 3B, 4, 6, 8 and 9) was engineered, which preferentially transduced human hepatocytes at higher efficiencies than rAAV8, rAAV3B and rAAV-DJ [50].

Notwithstanding the transduction efficiency seen with rAAV vectors, there are concerns regarding oncogenic integration. A particularly oncogenic site was identified in mice in a 6 kilobase region on chromosome 12 encoding various small nucleolar RNAs and microRNAs [51]. In humans, AAV2 clonal integration in known cancer driver genes was identified in various samples of human HCC [52]. For example, AAV2 was found to be integrated in the *TERT* promoter, which encodes telomerase reverse transcriptase, as well as in other genes in 11 of 193 HCCs analyzed. However, these findings were challenged on multiple bases by several groups [53,54], underscoring the uncertainty surrounding the potential for the insertional oncogenicity of AAV. In a Korean cohort, AAV-associated HCC was found to be very rare [55]; however, a recent genomic analysis of frozen hepatic parenchyma from 936 patients again demonstrated a correlation between AAV oncogenic insertion and the incidence of HCC in non-cirrhotic liver [56]. These results demonstrated a significant, albeit infrequent, risk of AAV-related insertional oncogenicity, although its significance as compared to wild-type exposure remains in question [57].

There have been various successful in vitro and in vivo applications of LTGT using AAV vectors. A murine model of Wilson’s disease demonstrated that a single injection of rAAV8, containing complementary DNA encoding *copper transporting ATPase 2*, normalized serum holoceruloplasmin levels and hepatic parenchymal copper levels for more than 6 months after administration [58]. Persistent expression of human porphobilinogen in a mouse model of acute intermittent porphyria reduced the frequency of biochemical attacks and the degree of neuropathy [59,60]. Intravenous delivery of propionyl-CoA carboxylase subunits via AAV8 decreased serum levels of toxins found in a murine model of propionyl acidemia [61]. Methylmalonic acidemia was corrected via the delivery of cDNA with an rAAV8 vector in a mouse model, preventing neonatal lethality [62]. In a murine model of phenylketonuria, the delivery of phenylalanine hydroxylase via an rAAV8 vector reduced serum phenylalanine levels [63]. In animal models of glycogen storage disease type Ia, with hypoglycemia, increased hepatic glycogen and hepatic steatosis, the rAAV8 delivery of *glucose-6-phosphatase* (*G6P*) resulted in the stable expression of G6P and a correction of the various defects in mice and canines [64,65]. An AAV8 vector was used to deliver *ATP binding cassette subfamily B member 4* (*ABCB4*) in a murine model of progressive familial intrahepatic cholestasis 3, leading to stable ABCB4 expression and the amelioration of the progression of liver fibrosis [66]. A canine study of hemophilia A, using AAV8 or AAV9 to deliver canine factor VIII resulted in the stable expression of factor VIII and a reduction in bleeding episodes [67]. It was observed that transgene expression unexpectedly declined after one year. A 10-year follow up study revealed stable expression of factor VIII with AAV genome integration and clonal expansion, but with no evidence of tumorigenesis or other adverse effects [68]. These animal studies emphasize the versatility of AAV vectors.

Given the safety, stability, lack of pathogenicity and low immunogenicity of AAV vectors, various clinical trials using rAAV vectors are underway. A clinical study in six patients with severe hemophilia B demonstrated that a peripherally administered AAV vector expressing human factor IX resulted in stable expression at 2–11% of normal levels, which was sufficient to ameliorate the bleeding diathesis [69]. Higher expression levels have been achieved more recently. As examples, a trial of hemophilia A using an AAV5 vector resulted in activity levels ranging from 19% to 164% one year after administration [45], and the use of a high-specific-activity factor IX variant resulted in expression levels ranging from 14% to 81% [70]. A three-year follow-up study revealed that two of the 15 patients in that trial had negligible expression of factor VIII, while others had sufficient expression and required minimal use of exogenous factor VIII [71]. While transgene expression may not be a permanent cure, the results are nonetheless encouraging.

A Phase I clinical trial for acute intermittent porphyria, using an rAAV2 vector delivering porphobilinogen deaminase, demonstrated safety but not metabolic correction at the doses tested, though varied outcomes were noted. Interestingly, two of eight patients were able to discontinue hematin treatment [72]. A Phase I/II clinical trial for homozygous familial hypercholesterolemia is currently underway (NCT02651675). Promising AAV-delivered agents currently in Phase III trials include AMT-061, a Padua variant Factor IX for hemophilia B (NCT03569891); fidanacogene elaparvovec, a high-activity factor IX gene for hemophilia B (NCT03587116); GS010, encoding the human wild-type ND4 protein for Leber hereditary optic neuropathy (NCT02652780, NCT02652767, NCT03293524); LYS-SAF302, encoding N-sulfoglucosamine sulfohydrolase for the treatment of mucopolysaccharidosis IIIA (NCT03612869); NSR-REP1, encoding REP1 for the treatment of choroideremia (NCT03496012); and valoctocogene roxaparvovec, encoding factor VIII for hemophilia A (NCT03370913; NCT03392974). Two AAV-based gene therapies that were recently approved by the FDA include Luxturna (voretigene neparvovec-rzyl) for the treatment of biallelic RPE65 mutation-associated retinal dystrophy [73] and Zolgensma (onasemnogene abeparvovec-xioi) for the treatment of spinal muscular atrophy [74].

These encouraging results showcase the promise of AAV vectors in LTGT, though the immunogenicity, oncogenicity, host AAV immunity and permanence of these vectors in humans remains a source of intense research. Indeed, the development of neutralizing antibodies against AAV vectors has been observed in clinical trials and has been correlated with a decrease in transduction efficacy [75,76,77,78,79], though this is not likely the main cause of the loss of transgene expression. The development of chimeric AAV vectors with increased cell type specificity and efficacy of transduction portends well for future clinical applications.

### 2.4. Simian Virus 40

Simian virus 40 (SV40) is a double-stranded DNA virus encoding two viral antigens, large (Tag) and small (tag) T cell antigens, as well as three structural proteins, VP1, VP2 and VP3 (Figure 1D) [80]. Recombinant SV40 (rSV40) has a transgene in place of the *Tag* gene, rendering it replication incompetent and much less immunogenic than wild-type SV40. A murine study of partially resected and regenerated liver showed that, when rSV40 was used to transduce mouse liver, transgene expression was comparable prior to resection and after regeneration. The results indicated that despite their replication incompetence, rSV40 vectors persist despite considerable cellular divisions and are highly stable [81]. In a murine model of Crigler–Najjar syndrome type I, intravenous administration of rSV40 encoding UGT1A1 resulted in stable expression of the transgene, normalization of alanine aminotransferase and bilirubin serum levels and normal liver histology [82]. However, ongoing concerns over its safety, due to the production of infectious wild-type SV40 particles in replication-defective SV40 packaging cell lines [83], have slowed the transition of this vector from preclinical research to clinical applications. More importantly, it has been observed that primary cell lines are immortalized in the presence of both large and small T antigens, that established cell lines are readily transformed and that tumors are induced in immunocompromised hamsters, events that do not transpire with the expression of individual T antigens [84]. Given these observations and the risk of vector-induced oncogenicity, there has been an effort to develop packaging cell lines with enhanced safety profiles. Recently, a cell line was developed that was able to produce high titers of replication-deficient SV40 particles with no detectable replication-competent revertant SV40 virions [85]. While the small size of SV40 precludes the packaging of larger transgenes, its high transduction efficiency, ability to produce high titers, versatile specificity, ease of administration, capacity to infect quiescent cells, low immunogenicity and high tolerogenicity make SV40 a potentially important tool for LTGT as the most immediate safety issues are addressed.

## 3. Non-Viral Vectors

### 3.1. Sleeping Beauty Transposon

Recognized as the first transposon-based system capable of gene transfer in vertebrate organisms, the *Sleeping Beauty* (*SB*) system, a transposon of the Tc1/mariner superfamily, uses a cut-and-paste mechanism for somatic gene transfer at TA dinucleotide sites and insertional mutagenesis [97,98]. *SB* displays considerable specificity at TA dinucleotide sites with additional potential specificity conferred by palindromic AT consensus sites [99], as well as DNA strand deformation [100]. A comparative study of *SB*, *piggyBac*, Moloney murine leukemia virus (MMLV) and HIV vector insertional site selection properties in CD4^+^ T cells found that *SB* had the highest probability of insertion into a safe harbor locus [101]. Additionally, there have been no reported preclinical *SB*-associated adverse effects [102,103,104,105]. The power of *SB* in identifying cancer driver genes is well known when used in systems with conditional transposase expression in various genetic backgrounds, including a recent murine study that used *SB* to identify drivers of erythroleukemia in mice [106]. While intentional insertional mutagenesis is a powerful application of *SB*, the risk of unintentional insertional mutagenesis appears to be mitigated by the specific conditions under which insertional mutagenesis occurs. For example, the use of transgenic mice constitutively expressing both transposon and transposase, as well as a specific genetic background (p53 +/−, etc.), are examples of conditions that impact the rate of unintentional insertional mutagenesis. It has been estimated that the risk of *SB* transposon remobilization leading to an adverse effect is about 10^−11^ [107].

*SB* has been successfully used in murine models of hemophilia A, hemophilia B, AAT, β-glucuronidase deficiency (mucopolysaccharidosis type VII), α-L-iduronidase deficiency (mucopolysaccharidosis type I) and hereditary tyrosinemia (*Fah* deficiency), with encouraging results [108,109,110]. It has been used for the in vivo overexpression of low-density lipoprotein and very low-density lipoprotein receptors, resulting in moderate reductions in plasma cholesterol and atherosclerosis in a murine model of familial hypercholesterolemia [111]. A study using a murine von Willebrand disease model found that *SB* transposon-mediated *von Willebrand factor* (*VWF*) gene delivery resulted in the long-term expression of supraphysiologic VWF levels [112]. While *SB* has been approved for certain clinical trials, delivery mechanisms to efficiently target livers in large animals are still under investigation. Further studies to fully characterize the inherent risk of transposase remobilization that could lead to genotoxic adverse reactions, including unintentional insertional mutagenesis, are warranted, notwithstanding the safety profile observed in preclinical studies.

### 3.2. piggyBac Transposon

The *piggyBac* DNA transposon was originally identified in baculovirus mutants as a host sequence in the cabbage looper *Trichoplusia ni* [113,114]. The *piggyBac* (*PB*) transposon–transposase system is known for its ability to insert at TTAA sites via a cut-and-paste mechanism similar to that of *SB* and to carry a larger transgene (as large as 100 kb) [115] as well as for its ability to precisely excise without leaving a genetic footprint [116]. *PB* has been used to transduce murine liver cells in vivo with the long-term stable expression of transgene [117,118]. *PB* has also been used to deliver cDNA encoding factor VIII in a murine model of hemophilia A, resulting in the stable expression of circulating factor VIII for over 300 days [119]. A codon-optimized *PB* transposase with enhanced activity was used in a murine model of hemophilia B with supraphysiologic factor IX expression for more than 12 months [120]. Regarding the risk of insertional mutagenesis, *PB* was tested in a sensitive HCC-prone murine model, with no tumor induction observed [120]. While *PB* demonstrated integration preference higher to that of *SB*, the overall integration profile of *SB* was more favorable to that of *PB* in primary human T lymphocytes [121]. The in vivo risk of insertional mutagenesis with *PB* remains to be determined.

### 3.3. Naked Nucleic Acids and Synthetic Delivery Vectors

Various methods for the delivery of DNA have been devised, including the injection of naked nucleic acids, hydrodynamic delivery, gene guns, sonoporation, electroporation and magnetofection, all used in small animals [122,123,124,125,126]. Since the half-life of intravenously delivered naked DNA is in the order of ~10 min in mice [127], the sequestration of DNA in a synthetic nanoparticle to protect from circulating endonucleases would increase time in circulation and ensure delivery to the target tissue. Indeed, various synthetic non-viral vectors have been developed, including liposomes [128,129], inorganic nanoparticles [130], polymers (such as linear polymers, branched polymers, dendrimers and polysaccharides) [131,132,133], polymersomes [134] and cell-penetrating peptides [135]. While there have been multiple successful applications of synthetic vectors, notably the conjugation of trivalent N-acetylgalactosamine to siRNA therapeutics (discussed below), various barriers to their widespread use remain. Such barriers include the potential for aggregation and disassembly in various physiologic conditions, potentially leading to microemboli in capillary beds, as well as clearance by macrophages [136] and effective nuclear delivery [137]. Further studies to identify the optimal synthetic vector(s) for LTGT are required, although their utility in FDA-approved therapeutics is encouraging.

## 4. RNA-Based Therapeutics as Gene Suppression Tools

### 4.1. Small Interfering RNAs

More recently, approaches to silence gene transcripts, thereby suppressing the translation of the respective gene products, have had promising clinical results in LTGT, with efficient non-viral delivery to hepatocytes. A small interfering RNA (siRNA), givosiran, bound to trivalent *N*-acetylgalactosamine specific to the asialoglycoprotein receptor on hepatocytes and delivered subcutaneously, targets and down regulates the translation of δ-aminolevulinic acid synthase 1 messenger RNA (mRNA). It was recently approved by the FDA to reduce the frequency of attacks of acute intermittent porphyria [138]. Another siRNA treatment, patisiran, which is delivered via liposome, was recently FDA approved for the treatment of transthyretin-related hereditary amyloidosis, a rare autosomal dominant disease which results in misfolded transthyretin and leads to diffuse polyneuropathy and nonischemic cardiomyopathy [139]. Alnylam, the producing firm, has several other products in their pipeline at various stages of clinical trials for PH1 (lumasiran) [140], hemophilia (fitusiran) [141] and HBV infection (ALN-HBV02) [142], among others. Another firm, Moderna, has several products in their pipeline at various stages of clinical investigation based on mRNA delivered via lipid nanoparticles. Their preclinical work includes mRNA encoding arginase 1 in a murine model of arginine deficiency, with moderate and stable expression levels [143]. Given the mechanism of siRNA-based gene therapy, there are questions regarding the optimal dosing required to achieve an acceptable level of therapeutic effect. For example, patisiran dosing is by IV every three weeks, and givosiran dosing is given subcutaneously every month. The frequency of dosing is one important consideration, in that siRNA-based therapies will require dosing for the rest of the patient’s life, whereas therapies administered via viral or non-viral vectors might not require repeated dosing.

### 4.2. microRNAs

MicroRNAs (miRNAs), which are short, non-coding, regulatory endogenous RNAs, about 19–25 nucleotides in length, have been implicated in hepatitis, cirrhosis and HCC [144,145,146]. The aforementioned deletion of miR-221/222 via adenoviral vector delivery resulted in attenuated hepatic fibrosis in a mouse model of NASH [28], while its reintroduction had the opposite effect. Another miRNA, miR-122, which is ubiquitously and specifically expressed in mammalian liver [147,148], appears to be involved in the pathogenesis of multiple liver diseases, from viral hepatitis via hepatitis C virus (HCV) [149], to hepatic steatosis [150], and HCC [151,152]. Antagomirs specific to miR-122 have been shown to improve NASH [150], as well as the inhibition of HCV viral replication and translation [149,153]. Lastly, miR-26a appears to be significantly down regulated in HCC [154]. Hepatic delivery of miR-26a via AAV in a murine model of HCC resulted in the suppression of tumorigenesis and the induction of tumor-specific apoptosis [154]. Though these studies showed efficacy of delivery using viral vectors, non-viral vectors have also been utilized to deliver miRNAs. Various non-viral vectors include liposomes, cell-derived membrane vesicles, gold nanoparticles and other inorganic material-based systems, as well as polymeric- and dendrimer-based vectors [155].

## 5. Genome Editing

The capacity for genetic engineering has enabled progressively more precise and specific applications through the recent creation of double-stranded breaks (DSBs) at specific target sequences. The monogenic nature of many inherited hepatic disorders makes endogenous DNA repair feasible with the evolution of more sophisticated gene editing techniques. Early developments of homologous recombination, single-stranded oligonucleotides and triplex DNA were important in foreshadowing the new era of gene editing for liver diseases [108,109,110].

### 5.1. Zinc Finger Nucleases

**Figure 2:**
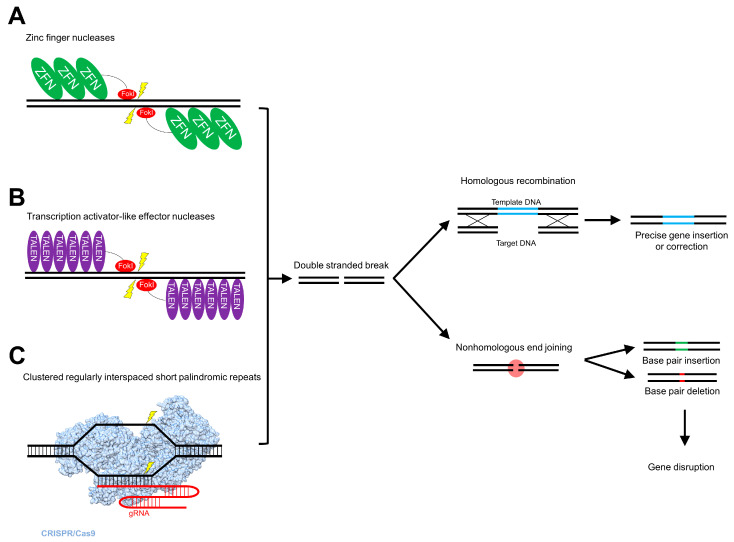
Recombinantly engineered nucleases utilize endogenous DNA repair mechanisms for genome editing. (**A**) Zinc finger nucleases (ZFNs), covalently bound to the FokI restriction enzyme nuclease domain, dimerize and generate double-stranded breaks (DSBs) in target DNA. (**B**) Similarly, transcription activator-like effector nucleases (TALENs) bind to target DNA, and the FokI nuclease domain generates DSBs. (**C**) Clustered regularly interspaced short palindromic repeats (CRISPR) bound to CRISPR-associated protein 9 (Cas9) contain a guide RNA (gRNA) (PDB ID: 5U0A) [165]. Through hybridization of the gRNA to the target region, DSBs are generated at specific sites, with subsequent insertion of the donor DNA. DSBs are a substrate for two endogenous DNA repair mechanisms; homologous recombination (HR) and nonhomologous end joining (NHEJ). HR results in the integration of donor DNA with precision and fidelity, whereas NHEJ results in insertions and deletions at the DSB cleavage site of varying base pair lengths, thus resulting in gene disruption, without the insertion of donor DNA. The inability to favor one mechanism over another results in off-target effects and unintended mutations, insertions and deletions inherent to these endogenous mechanisms.

Zinc finger nucleases (ZFNs) are recombinantly engineered endonucleases comprising the DNA-binding domain of zinc finger proteins fused to the C-terminal DNA cleavage domain of the *Flavobacterium okeanokoites Fok*I restriction enzyme (Figure 2A) [156]. Their adaptability and versatility have been demonstrated in numerous successful applications in LTGT, most notably the in vivo correction of factor IX deficiency in a murine model of hemophilia B [157]. The combination of ZFNs with viral vectors, specifically recombinant adeno-associated virus (rAAV), has led to the correction of hemophilia A and B in adult mice, as well as lysosomal enzyme deficiencies in Fabry and Gaucher diseases, along with Hurler and Hunter syndromes [158,159]. Though ZFNs are considered a feasible component of the LTGT armamentarium, there continues to be concern over the frequency of off-target cleavage events.

### 5.2. Transcription Activator-Like Effector Nucleases

Transcription activator-like effector nucleases (TALENs) are recombinantly engineered nucleases comprising the DNA recognition domain of the *Xanthomonas* transcription activator-like effector fused to the DNA cleavage domain of the *Fok*I restriction enzyme (Figure 2B). In LTGT, murine hepatocytes were engineered using TALENs to be deficient in *UGT1A1*, thus deriving an in vitro model of Crigler–Najjar syndrome type 1 [160]. An additional study found that hepatitis C virus (HCV) entry into Huh-7.5 cells was significantly impaired in a claudin-1-dependent manner when *diacylglycerol acyltransferase-1* expression was silenced using TALENs [161]. The large size of TALENs and epigenetic modifications impairing access to the desired chromosomal site are significant challenges for use in a clinical setting.

### 5.3. Clustered Regularly Interspaced Short Palindromic Repeats

Bacteria and archaea possess adaptive immune defense mechanisms such as clustered regularly interspaced short palindromic repeats (CRISPR), which are highly conserved, repeating and non-contiguous loci. The CRISPR loci are transcribed as precursor CRISPR RNAs (pre-crRNAs), which bind to *trans*-activating crRNA to form duplex RNAs that are cleaved by one of the *CRISPR-associated* (*Cas*) genes, namely *Cas9*, that encodes a double-stranded RNA-specific ribonuclease RNase III (Figure 2C) [162]. The cleavage product of Cas9-duplex RNA interaction is a single-stranded guide RNA (gRNA), which binds to Cas9. As with ZFNs and TALENs, CRISPR/Cas9 cleaves DNA to form DSBs, which are then repaired by homologous recombination (HR) or nonhomologous end joining (NHEJ) [163,164].

CRISPR/Cas9 has been used in hereditary tyrosinemia to correct the *Fah* mutation in a mouse model [166]. Murine AAV vector delivery of CRISPR/Cas9 targeted to the low-density lipoprotein receptor induced severe hypercholesterolemia and atherosclerosis [167]. An in vitro study using induced pluripotent stem cell-derived hepatocyte-like cells (iHLCs) with an engineered deactivated *Arg1* locus (which encodes arginase-1) demonstrated the utility of CRISPR/Cas9 to reincorporate excised *Arg1* exons and thus reconstitute arginase function in arginase-1 deficiency [168]. Recently, human AAT was somatically incorporated into mouse liver using CRISPR/Cas9 with an adenoviral vector to achieve stable expression for >200 days [169]. Lastly, skin fibroblasts from a patient with PH1 were reprogrammed ex vivo into iHLCs [170]. The PH1-iHLCs containing the defective liver-specific enzyme AGT underwent CRISPR/Cas9 gene delivery of an *AGXT* therapeutic minigene, inserting into a safe harbor locus without detectable off-target insertions [171].

Several more recent LTGT applications demonstrate the ability of the CRISPR/Cas9 system to eradicate chronic HBV infection in duck hepatocytes, via the targeted inhibition of covalently closed circular DNA (cccDNA) [172]. Another in vitro study using human HepG2 cells demonstrated the efficient degradation of HBV cccDNA using *Streptococcus pyogenes* (Sp) and *Streptococcus thermophilus* (St) Cas9 [173]. HBV genome inhibition in mice was recently reported using *Staphylococcus aureus* Cas9 delivered using an AAV vector [174]. Lastly, a clinical trial of 10 patients with HCC currently underway in China uses CRISPR to engineer autologous *PD-1* knockout T lymphocytes ex vivo. Following transcatheter arterial chemoembolization to obliterate the vascular supply, percutaneously administered autologous *PD-1* knockout cells are delivered to the tumor periphery to ascertain the response rate and progression-free survival (NCT04417764). This approach, if successful, has exciting implications in the treatment of HCC.

Based on the observation of off-target insertions with CRISPR-based genome editing, there has been significant discussion regarding the risk of insertional mutagenesis and the development of cancer. The generation of DSBs by conventional CRISPR-based methods leads to the activation of *p53* [175], which can interfere with CRISPR genome editing. There is concern that the selection of cells modified by CRISPR could include cells with *p53* mutations, posing a significant risk of cancer. Of note, a murine study demonstrated the direct mutation of *p53* and *Pten* genes, resulting in the induction of hepatocellular carcinoma (HCC) [176]. The potential risk of cancer has led to the development of novel techniques based on CRISPR that avoid the generation of DSBs.

**Figure 3:**
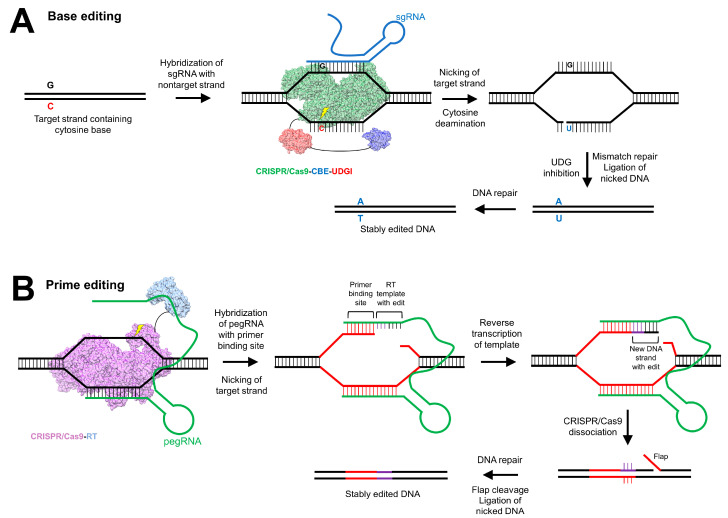
Base editing and prime editing use catalytically impaired Cas9 nuclease for single-strand nicking. (**A**) Base editing utilizes a catalytically impaired Cas9 nuclease (PDB ID: 5U0A) [165] fused with a cytosine base editor (CBE) (PDB ID: 1MQ0) [181] (or, alternatively, an adenine base editor), as well as a uracil DNA glycosylase inhibitor (UDGI) (PDB ID: 1UGH) [182]. The CRISPR/Cas9-CBE-UDGI also contains a single-guide RNA (sgRNA), which hybridizes with a target strand containing a cytosine–guanine base pair. The sgRNA binds to the nontarget strand containing the guanine base, and the Cas9 nuclease performs a single-stranded break (“nicking”) of the target strand on the 5′ end of the target cytosine base. The CBE domain catalyzes cytosine deamination to generate uracil on the target DNA strand, which is a substrate for the endogenous base excision repair (BER) mechanism. The UDGI domain suppresses BER, favoring mismatch repair to replace guanine on the nontarget strand with adenine. The nicked target strand is ligated, and through DNA repair, uracil on the target strand is replaced with thymidine, thus generating an adenine–thymidine base pair and stably edited DNA. (**B**) Prime editing uses a catalytically deficient Cas9 capable only of nicking (PDB ID: 5U0A) [165], fused to the Moloney murine leukemia virus reverse transcriptase (PDB ID: 1MML) [183], and bound to a prime editing guide RNA (pegRNA) that targets the region of interest in genomic DNA. After cleavage of the target strand, the pegRNA hybridizes with the primer binding site. Adjacent to this site on the pegRNA is the reverse transcriptase (RT) template with the gene editing template. The RT motif reverse transcribes the target strand using the pegRNA as a template. Once the pegRNA dissociates, the 3′ end with the reverse transcribed insertion binds to the nontarget DNA with a mismatch at the gene editing site. There is a flap at the 5′ end of the nicked strand that is cleaved off, and the nicked ends are subsequently ligated. The mismatched base pairs undergo DNA repair to generate stably edited DNA.

An approach called base editing utilizes a catalytically impaired Cas9 fused to a base modification enzyme (a cytosine base editor that generates uracil, or an adenine base editor that generates inosine) as well as to a uracil DNA glycosylase inhibitor (Figure 3A) [177,178]. The fusion protein is bound to a guide RNA that displaces a small segment in the target region and uses single-stranded DNA as a substrate to generate a nick in the non-edited strand. To prevent the endogenous base excision repair mechanism from reversing the engineered base modification, the uracil DNA glycosylase inhibitor blocks the action of uracil N-glycosylase to remove the base from the DNA backbone. A recent exciting application of this approach was the correction of murine phenylketonuria via the AAV delivery of CRISPR/Cas9 base editing agents [179]. A newer approach, termed prime editing, utilizes a catalytically impaired Cas9 fused to reverse transcriptase to allow for direct copying of genetic information into the target site without the induction of DSBs and the potential for genomic mutations (Figure 3B) [180]. Such technical advances, which appear to mitigate the risk of off-target insertions and deletions associated with DSBs, are exciting breakthroughs that will enhance the utility of genome editing approaches.

## 6. Future Perspectives

The exciting prospects for liver-targeted gene therapy in the age of precise targeting and genome editing, with an ever-expanding array of therapeutics in clinical trials and on the market (summarized in Table 1), hold special promise for the treatment of monogenic metabolic disorders. As outlined above, one overarching principle is mitigating the risk of insertional mutagenesis, as the preclinical success of novel approaches may be masked by inherent oncogenic risk. Robust studies should be carried out that comprehensively characterize the oncogenic risk of all gene therapy approaches that involve genomic integration. Closely related to this is the specificity of various approaches; for example, the specificity of CRISPR is a topic of ongoing debate, and while approaches such as base editing and prime editing provide an opportunity to optimize specificity, preclinical studies using these two approaches are scant. Furthermore, and this is especially true for viral vectors, innate and adaptive immune responses against both the vector and the payload need to be taken into account, as work in preclinical animal models often does not translate to clinical trials. The employment of strict exclusion criteria to account for seropositive patients likely to have a suboptimal response will continue and will likely broaden to multifactorial exclusion criteria based on genotypic factors. Payload size is another rate-limiting step in the further development of various viral and non-viral vectors, as many vectors are only capable of delivering transgenes less than 10 kb. Lastly, given the high cost of research and development for many of these therapeutics and their exorbitant prices when they reach the market, regulatory frameworks from both the FDA and the Europe Medicines Agency (EMA) aim to shorten the time of development and speed up the time to clinical trials. This will undoubtedly reduce development costs and the potential financial burden to the healthcare system. In summary, the optimization of viral and non-viral vectors to improve specificity and mitigate oncogenicity, the more complete characterization of vector safety profiles, the broader screening of patients for clinical trials and the development of regulatory frameworks to expedite these products to market with greater safety and reduced cost are critical factors in the application of liver-targeted gene therapy to a myriad of disorders.

## 7. Conclusions

Our expanding arsenal of techniques for gene editing and delivery systems herald a promising outlook for LTGT. Given the multitude of monogenic metabolic disorders that have been clinically and mechanistically described, the feasibility that LTGT could provide a cure to such disorders is potentially in reach. While many obstacles remain, particularly the additional characterization of safety and efficacy between the various systems, optimal delivery routes, circumventing vector immunogenicity and enhancing cell type specificity, the potential of LTGT to correct genetic deficiencies and improve quality of life in patients with disabling metabolic disorders is very promising. Now that many of these therapies translate to clinical trials, ensuring the safety, tolerogenicity and robust monitoring of adverse reactions will be of paramount importance. The combination of cutting-edge genome editing approaches with viral and non-viral vectors is a powerful approach that can transform the practice of medicine and cure many monogenic ailments. Future refinements of these methods could target polygenic disorders, and their ability to regulate gene expression could yield more effective therapeutics for a wide range of diseases. As these systems mature with improved safety and efficiency, the diversity of clinical trials will undoubtedly blossom and grow.

## Figures and Tables

**Table 1:** Summary of preclinical and clinical trials of liver targeted gene therapy.

Vector	Payload	Disorder	Preclinical Results/Clinical Trial Endpoints	Clinical Trial/Phase	References
Retroviral	*Factor VIII*	Hemophilia A	Physiologic FVIII levels (mice)		[6]
Lentiviral	*Factor IX*	Hemophilia B	Supraphysiologic FIX levels (nonhuman primates)		[20]
Lentiviral	*Factor IX*	Hemophilia B	Sustained FIX expression, prevention of NAb induction (mice)		[21]
Lentiviral	CRISPR/Cas9	Hepatitis B	Inhibition of HBV replication in vivo (mice) and in vitro		[22,23]
Lentiviral	*Factor IX*	Hemophilia B	Ex vivo stem cell gene correction and autologous stem cell transplant	NCT03961243/Phase I	
Adenoviral	*UGT1A1*	Crigler-Najjar type I	Correction of hyperbilirubinemia (rats)		[26]
Adenoviral	Human *aquaporin 1*	Estrogen-induced cholestasis	Lower serum bile salt concentration, improved biliary output (rats)		[27]
Adenoviral	miR-221/222	NASH	Decreased hepatic fibrosis (mice)		[28]
HDAd/*Sleeping Beauty*	*Factor IX*	Hemophilia B	Stable FIX expression (dogs)		[39,40]
HDAd	*AGT*	Primary hyperoxaluria 1	Stable transgene expression, decreased hyperoxaluria (mice)		[41]
HDAd	*AAT*	AAT deficiency	Stable AAT expression (nonhuman primates)		[32]
AAV	*Factor IX*	Hemophilia B	FIX expression decreased by 92% following 2/3 hepatectomy (mice)		[48]
AAV	*Factor IX*	Hemophilia B	Robust FIX expression levels (mice)		[49]
AAV	*Copper transporting ATPase 2*	Wilson’s disease	Normalized serum holoceruloplasmin levels and hepatic parenchymal copper levels (mice)		[58]
AAV	Human *porphobilinogen*	Acute intermittent porphyria	Reduced frequency of biochemical attacks, degree of neuropathy (mice)		[59,60]
AAV	*Propionyl-CoA*	Propionyl acidemia	Decreased serum toxin level (mice)		[61]
AAV	Human *methylmalonyl-CoA mutase*	Methylmalonic acidemia	Correction of acidemia, prevention of murine neonatal lethality		[62]
AAV	*Phenylalanine hydroxylase*	Phenylketonuria	Reduced serum phenylalanine levels (mice)		[63]
AAV	*Glucose-6-phosphatase*	Glycogen storage disease type Ia	Stable G6P expression, correction of hypoglycemia, normalized hepatic glycogen and reduced hepatic steatosis (mice, dogs)		[64,65]
AAV	*ABCB4*	Progressive familial intrahepatic cholestasis 3	Stable ABCB4 expression, reduced progression of liver fibrosis (mice)		[66]
AAV	*Factor VIII*	Hemophilia A	Stable FVIII expression (dogs)		[67,68]
AAV	*Factor IX*	Hemophilia B	Stable FIX expression	NCT00979238/Phase I	[69]
AAV	*Factor VIII*	Hemophilia A	Stable FVIII expression	NCT02576795/Phase I/II	[45]
AAV	*Factor IX*	Hemophilia B	Stable FIX expression	NCT02484092/Phase II	[70,71]
AAV	*Porphobilinogen deaminase*	Acute intermittent porphyria	Safety, not metabolic correction at doses tested, varied results	NCT02082860/Phase I	[72]
AAV	*LDLR*	Homozygous familial hypercholesterolemia	Improvement of lipid profile	NCT02651675/Phase I/II	
AAV	Padua variant *factor IX*	Hemophilia B	Supraphysiologic FIX expression level	NCT03569891/Phase III	
AAV	Fidanacogene elaparvovec (high activity factor IX)	Hemophilia B	Supraphysiologic FIX expression level	NCT03587116/Phase III	
AAV	GS010 (human wild-type *ND4*)	Leber hereditary optic neuropathy	Recovery of vision	NCT02652780 NCT02652767 NCT03293524, all Phase III	
AAV	LYS-SAF302 (*N-sulfoglucosamine sulfohydrolase*)	Mucopolysaccharidosis IIIA	Improvement or stabilization of neurodevelopmental state	NCT03612869/Phase III	
AAV	NSR-REP1 (*REP1*)	Choroideremia	Improvement in best corrected visual acuity	NCT03496012/Phase III	
AAV	Valoctocogene roxaparvovec (*factor VIII*)	Hemophilia A	Improvement in FVIII median activity	NCT03370913 NCT03392974, both Phase III	
AAV	Voretigene neparvovec-rzyl (*RPE65*)	Biallelic RPE65 mutation-associated retinal dystrophy	Improvement in multi-lumen mobility test scores	FDA approved	[73]
AAV	Onasemnogene abeparvovec-xioi (*SMN*)	Spinal muscular atrophy	Prevention of death and permanent breathing support	FDA approved	[74]
SV40	*UGT1A1*	Crigler-Najjar type I	Normalization of murine ALT and bilirubin serum levels, liver histology		[82]
*Sleeping Beauty*	*Factor VIII*	Hemophilia A	Stable FVIII expression		[108,109,110]
*Sleeping Beauty*	*Factor IX*	Hemophilia B	Stable FIX expression		[108,109,110]
*Sleeping Beauty*	*AAT*	AAT deficiency	Stable AAT expression (mice)		[108,109,110]
*Sleeping Beauty*	*β-glucuronidase*	Mucopolysaccharidosis type VII	Stable β-glucuronidase expression (mice)		[108,109,110]
*Sleeping Beauty*	*α-L-iduronidase*	Mucopolysaccharidosis type I	Stable α-L-iduronidase expression (mice)		[108,109,110]
*Sleeping Beauty*	*Fah*	Hereditary tyrosinemia	Stable Fah expression (mice)		[108,109,110]
*Sleeping Beauty*	*LDLR*, *VLDLR*	Familial hypercholesterolemia	Moderate reduction in plasma cholesterol and atherosclerosis (mice)		[111]
*Sleeping Beauty*	*von Willebrand factor*	von Willebrand disease	Supraphysiologic VFW levels (mice)		[112]
*piggyBac*	*Factor VIII*	Hemophilia A	Stable FVIII expression		[119]
*piggyBac*	*Factor IX*	Hemophilia B	Stable FIX expression		[120]
Non-viral (trivalent N-acetylgalactosamine)	Givosiran (siRNA)	Acute intermittent porphyria	Silences *δ-aminolevulinic acid synthase 1* mRNA, reduces AIP attack frequency	FDA approved	[138]
Non-viral (liposomes)	Patisiran (siRNA)	Transthyretin-related hereditary amyloidosis	Improvement in polyneuropathy	FDA approved	[139]
Non-viral (trivalent N-acetylgalactosamine)	Lumasiran (siRNA)	Primary hyperoxaluria 1	Decreased hyperoxaluria by silencing glycolate oxidase	NCT02706886/Phase I/II	[140]
Non-viral (trivalent N-acetylgalactosamine)	Fitusiran (siRNA)	Hemophilia A & B	Reduces bleeding instances by silencing antithrombin	NCT03417245/Phase III	[141]
Non-viral (trivalent N-acetylgalactosamine)	ALN-HBV02 (siRNA)	Hepatitis B	Reduction in HBV surface antigen levels	NCT02826018/Phase I	[142]
Non-viral (lipid nanoparticle)	*Arginase 1*	Arginine deficiency	Stable, moderate arginase 1 expression (mice)		[143]
Non-viral (SQ injection)	miR-122 antagomir	NASH	Improvement in hepatic steatosis and reduction in plasma cholesterol (mice)		[150]
Non-viral (SQ injection)	miR-122 antagomir	Hepatitis C	Inhibition of viral replication and translation in vitro		[149,153]
AAV	miR-26A	Hepatocellular carcinoma	Suppression of tumorigenesis and induction of tumor-specific apoptosis (mice)		[154]
AAV	Factor VIII, ZFN	Hemophilia A	Stable expression of FVIII (mice)		[157]
AAV	Factor IX, ZFN	Hemophilia B	Stable expression of FIX (mice)		[158,159]
AAV	Human *α-galactosidase A*, ZFN	Fabry disease	Stable expression of α-galactosidase A (mice)		[159]
AAV	*Acid β-glucosidase*, ZFN	Gaucher disease	Stable expression of acid β-glucosidase (mice)		[159]
AAV	*Iduronate-2 sulfatase*, ZFN	Hunter syndrome	Stable expression of iduronate-2 sulfatase (mice)		[159]
AAV	*α-L-iduronidase*, ZFN	Hurler syndrome	Stable expression of α-L-iduronidase (mice)		[159]
Lentiviral	TALENs targeting *diacylglycerol acyltransferase-1*	Hepatitis C	Viral entry impaired in a claudin-1 dependent manner in vitro		[161]
Non-viral (ssDNA oligonucleotides)	*Fah*, CRISPR/Cas9	Hereditary tyrosinemia	Correction of *Fah* mutation (mice)		[166]
AAV	CRISPR/Cas9	Induction of severe hypercholesterolemia and atherosclerosis	Via mutation of *LDLR* (mice)		[167]
Adenoviral	AAT, CRISPR/Cas9	AAT deficiency	Somatic murine hepatic incorporation and stable expression of AAT		[168]
AAV	CRISPR/Cas9	Hepatitis B	Inhibition of genome replication in mice		[173]
Non-viral (DNA plasmids)	CRISPR/Cas9	Induction of hepatocellular carcinoma	Via mutation of *p53* and *Pten* (mice)		[176]
None	*Ex vivo PD-1* knockout in T lymphocytes engineered via CRISPR	Hepatocellular carcinoma	Tumor response rate, progression-free survival	NCT04417764	
AAV	CRISPR/Cas9	Phenylketonuria	Correction of *PKU* mutation via base editing (mice)		[179]

## Abbreviations

AAT	α-1 antitrypsin
AAV	adeno-associated virus
AAVR	adeno-associated virus receptor
AGXT	alanine-glyoxylate aminotransferase
Arg1	arginase-1
Cas	CRISPR-associated gene
cccDNA	covalently closed circular DNA
CRISPR	clustered regularly interspaced short palindromic repeats
DNA	deoxyribonucleic acid
DSB	double-stranded break
HBV	hepatitis B virus
HCC	hepatocellular carcinoma
HCV	hepatitis C virus
HDAd	helper-dependent adenovirus
HIV	human immunodeficiency virus
HR	homologous recombination
iHLC	induced hepatocyte-like cell
LTGT	liver-targeted gene therapy
MLV	murine leukemia virus
M-MLV	Moloney murine leukemia virus
NASH	non-alcoholic steatohepatitis
NHEJ	nonhomologous end joining
pegRNA	prime editing guide RNA
PH1	primary hyperoxaluria type 1
rAAV	recombinant adeno-associated virus
rAd	recombinant adenovirus
RNA	ribonucleic acid
rSV40	recombinant simian virus 40
SB	Sleeping Beauty
Sp	Streptococcus pyogenes
St	Streptococcus thermophilus
SV40	simian virus 40
TALEN	transcription activator-like effector nuclease
Tag	T cell antigen
TERT	telomerase reverse transcriptase
UGT1A1	uridine diphosphate glucuronosyltransferase family 1 member A1
ZFN	zinc-finger nuclease
